# Effects of Mindfulness Training on Sleep Problems in Patients With Fibromyalgia

**DOI:** 10.3389/fpsyg.2018.01365

**Published:** 2018-08-03

**Authors:** Alberto Amutio, Clemente Franco, Laura C. Sánchez-Sánchez, María del C. Pérez-Fuentes, José J. Gázquez-Linares, William Van Gordon, María del M. Molero-Jurado

**Affiliations:** ^1^Department of Social Psychology and Methodology of the Behavioral Sciences, University of the Basque Country (UPV/EHU), Bilbao, Spain; ^2^Department of Psychology, University of Almería, Almería, Spain; ^3^Department of Psychology, Universidad Autónoma de Chile, Santiago, Chile; ^4^Centre for Psychological Research, University of Derby, Derbyshire, United Kingdom

**Keywords:** fibromyalgia syndrome, mindfulness, sleep disorders, sleep quality, second-generation mindfulnessbased interventions

## Abstract

Fibromyalgia syndrome (FMS) is a complex psychosomatic pain condition. In addition to generalized pain and various cognitive difficulties, new FMS diagnostic criteria acknowledge fatigue and sleep problems as core aspects of this condition. Indeed, poor sleep quality has been found to be a significant predictor of pain, fatigue, and maladaptive social functioning in this patient group. While there is promising evidence supporting the role of mindfulness as a treatment for FMS, to date, mindfulness intervention studies have principally focused on dimensions of pain as the primary outcome with sleep problems either not being assessed or included as a secondary consideration. Given the role of sleep problems in the pathogenesis of FMS, and given that mindfulness has been shown to improve sleep problems in other clinical conditions, the present study explored the effects of a mindfulness-based intervention known as *Flow Meditation* (*Meditación-Fluir*) on a range of sleep-related outcomes (subjective insomnia, sleep quality, sleepiness, and sleep impairment) in individuals with FMS. Adult women with FMS (*n* = 39) were randomly assigned to the 7 weeks mindfulness treatment or a waiting list control group. Results showed that compared to the control group, individuals in the mindfulness group demonstrated significant improvements across all outcome measures and that the intervention effects were maintained at a 3 month follow-up assessment. The *Meditación-Fluir* program shows promise for alleviating sleep problems relating to FMS and may thus have a role in the treatment of FMS as well as other pain disorders in which sleep impairment is a central feature of the condition.

## Introduction

Fibromyalgia syndrome (FMS) is a chronic pain condition characterized by localized pain spots as well as prolonged generalized body pain. Other typical symptoms include fatigue, trouble sleeping, headaches, numbness in the hands and feet, depression, anxiety and decreased tolerance to exercise (Staud et al., 2001; Costa et al., 2017; Sosa-Reina et al., 2017). FMS affects around 3% of the general population, with a seven-to-one higher prevalence in women aged 20–50 years old versus men of the same age (Prados and Miró, 2012; Lauche et al., 2013; Feliu-Soler et al., 2016). Studies demonstrate a deterioration in quality of life in individuals suffering from FMS compared to those with other rheumatic or autoimmune diseases (Hoffman and Dukes, 2008; González et al., 2010). FMS has a strong negative impact on family and social engagement and imposes substantial healthcare and social services costs (Rivera et al., 2006; Langhorst et al., 2013; Van Gordon et al., 2016).

In 2010, the American College of Rheumatology proposed a new set of diagnostic criteria for FMS replacing the traditional 1990 classification (Wolfe et al., 1990). In addition to including generalized pain and various cognitive difficulties, the new criteria acknowledged fatigue and sleep problems as core aspects of the condition (Wolfe et al., 2010; Schmidt et al., 2011). Research has shown that sleep impairment can lower the threshold for pain as well as trigger musculoskeletal pain and emotional distress (Prados and Miró, 2012; Becker et al., 2018). Indeed, in a study of 101 FMS patients, 99% exhibited poor sleep quality that was found to be a significant predictor of pain, fatigue, and maladaptive social functioning (Miró et al., 2011). Research also shows that sleep quality moderates the relationship between affect and pain in patients with FMS, and that a high quality of sleep thus increases patients’ tolerance to pain episodes. Similarly, it has been shown that sleep quality is a significant mediator in the relationship between pain and anxiety or depression in FMS patients (Miró et al., 2011; Li et al., 2018). Furthermore, both poor quality sleep and pain can elicit catastrophizing thought and behavior patterns which in turn lead to the impairment of pain coping cognitive strategies (Prados and Miró, 2012; Di Tella et al., 2018). Thus, sleep problems play an important role in the exacerbation of FMS symptoms and increase the likelihood of both depression and impaired physical and psychological functioning in this patient group (Bigatti et al., 2008; González et al., 2010; Finan et al., 2013; Galvez-Sánchez et al., 2018).

Recommended treatments for FMS include anti-inflammatories and antidepressants as well as other medication to target mechanisms of pain (Crofford and Appleton, 2000; Crofford, 2008). However, sleep disturbances are one of the most common residual symptoms of antidepressant medication (ADM), and are associated with (amongst other things) exacerbating and perpetuating mood disturbance, poor quality of life, and reductions in mindfulness levels in FMS patients (Modrego et al., 2016; Li et al., 2018). In addition, sleep studies using polysomnography have shown that ADMs can disrupt sleep continuity and increase the number of awakenings during periods of sleep (Britton et al., 2012).

Consequently, more effective treatment strategies for FMS are likely those that use pharmacotherapy in moderation and integrate non-pharmacological approaches such as mindfulness, relaxation therapy, cognitive-behavioral therapy, physical exercise, and/or psycho-education (Glombiewski et al., 2010; Parra and Latorre, 2013; Van Gordon et al., 2016). Given the importance of sleep in the physiopathology of fibromyalgia, mindfulness in particular warrants further investigation because: (i) it has been shown to improve sleep quality in a range of pathologies (Shonin et al., 2015), and (ii) improvements in quality of life in individuals with FMS after receiving a mindfulness-based intervention known as *Meditation Awareness Training (MAT)* appear to be linked to increases in sleep quality (Van Gordon et al., 2016, 2017). Furthermore, mindfulness has also been shown to be beneficial in targeting certain symptoms of FMS, such as depression, anxiety, anger, and poor quality of life (Grossman et al., 2007; Sephton et al., 2007; Schmidt et al., 2011; Amutio et al., 2015a; Kanen et al., 2015). In line with these findings, a correlational study of FMS patients (*n* = 4,986) showed that trait mindfulness was negatively associated with symptom severity (Jones et al., 2015). However, despite these promising findings, to date studies evaluating the effectiveness of mindfulness as a treatment for FMS have principally focused on dimensions of pain as the primary outcome with sleep problems either not being assessed or included as a secondary consideration in conjunction with other outcomes relevant to the condition (Schmidt et al., 2011; Cash et al., 2015; Martínez et al., 2016).

Given the role of sleep problems in the pathogenesis of FMS, the lack of conclusive data for extant pharmacological and non-pharmacological FMS treatments, and the fact that mindfulness has been shown to improve sleep problems and fatigue in various other clinical and non-clinical conditions (Britton et al., 2012; Rimes and Wingrove, 2013; Hülsheger et al., 2015; Kanen et al., 2015), there is a need to specifically assess the effects of mindfulness on a range of sleep-related outcomes in individuals with FMS. Thus, the purpose of the present study was to evaluate the effects of a mindfulness-based intervention known as *Meditación- Fluir -Flow Meditation –* (Franco, 2009) on subjective insomnia, sleep quality, sleepiness, and sleep impairment in individuals with FMS. It was hypothesized that compared to a waitlist control group, individuals with FMS that completed the mindfulness intervention would demonstrate significant improvements in the aforementioned variables.

## Materials and Methods

A randomized controlled trial was conducted to investigate the effects of mindfulness training on subjective insomnia, sleep quality, sleepiness, and sleep impairment in individuals with FMS. Assessments were taken at pre-test, post-test, and 3 month follow-up phases and the intervention group was compared with a waiting list control group.

### Participants

To be eligible for the study, participants had to be: (i) able to prove a current diagnosis of FMS (e.g., via a letter from a doctor of pain consultant), (ii) female, (iii) aged 18–70 years, and (iv) not currently undergoing mindfulness training and/or formal psychotherapy (stable prescription medication was permitted for both the intervention and control group). All participants were recruited from the *Asociación de Fibromialgia de Almería* (AFIAL) and were informed that they would be undertaking a program of training in mindfulness for individuals with FMS. A total of 39 eligible individuals were recruited into the study and random assignment was employed to allocate participants to the intervention (*n* = 20) or control group (*n* = 19). More specifically, ballots, concealing the numbers 1 (control group) or 2 (intervention group), were placed in equal number into an urn and each participant extracted one ballot from it. Randomization was conducted by a researcher not participating in the study and participants completed baseline assessments prior to allocation. Due to a logistical constraint, a sample size calculation was not conducted because sample size was governed by the maximum number of eligible female participants – based at the aforementioned regional FMS association – that were willing to participate in the study at the time of enrolment.

### Procedure

A mindfulness training program called *Flow Meditation* (*Meditación-Fluir* – Franco, 2009) was administered to the intervention group using 2 h weekly group sessions for a period of 7 weeks. The training program included: (i) mindfulness exercises from Kabat-Zinn’s (1990) stress-reduction program, (ii) mindfulness techniques used in acceptance and commitment therapy (Hayes et al., 1999; Wilson and Luciano, 2002; Carrascoso, 2006), and (iii) exposure to and debate on metaphors and exercises used in Zen (Deshimaru, 2006) and Vipassana meditation (Hart, 1994) which promote values such as acceptance, forgiveness, and non-identification with mental events. The effectiveness of *Meditación-Fluir* has been tested in various treatment studies with acceptable size effects (Amutio et al., 2015a; Franco et al., 2017, 2016). The program includes existential, ethical, and spiritual aspects and values, and thus conforms to Van Gordon et al.’s (2015) criteria for *Second Generation Mindfulness-Based Interventions (SG-MBIs)*. The program was facilitated by an instructor with extensive experience in both the practice and teaching of mindfulness meditation techniques.

The main purpose of *Meditación-Fluir* is for participants to learn to allow their thoughts to flow, without trying to modify them or interfere with them. The intervention is not concerned with teaching participants not to think about anything, but seeks to offer an alternative to conditioned automatic ways of reacting to inner and outer experiences. Each weekly session is structured as follows:

(1) Discussion and feedback on the mindfulness meditation exercises practiced during the previous week.
(2) Ten minutes guided mindful body-scan.
(3) Presentation of the various metaphors and exercises corresponding to each session.
(4) Practice of full awareness of breathing for 30 min.

Both during the active treatment and follow-up phases, participants were requested to undertake mindful body-scan and full awareness of breathing exercises on a daily basis at home for 10 and 30 min, respectively. Furthermore, participants were also asked to keep a daily record of their level of engagement with at-home practices by means of a register sheet especially designed for this purpose.

### Ethical Considerations

All participants provided informed consent and the study was approved by the Bioethics Committee of the University of Almeria, Spain. The registered data for each of the instruments was alphanumerically coded, ensuring confidentiality and anonymity, in order to comply with the Personal Data Protection Act by the Ethics Committee for Research related to Human Beings (CEISH). International guidelines for studies with human subjects described in the Nuremberg Code and in the Declaration of Helsinki were applied. After completion of all assessment phases, the mindfulness training course was offered to control group participants.

### Measures

The following measures were administered pre- and post-intervention and at 3 months after the final group session in week seven:

(i) *Athens Insomnia Scale* (Soldatos et al., 2000). This scale enables the subjective presence of insomnia to be identified based on the diagnostic criteria of the Classification of Mental and Behavioral Disorders (ICD-10). The scale comprises eight items and the first five items evaluate difficulty inducing sleep, waking up at night, waking up early, and total sleep quality. The last three items explore the daytime consequences of sleep problems including sleepiness, reduced well-being and poor functioning. Higher scores correspond to more severe sleep impairment and a score over five indicates the presence of insomnia. In the present study, the Spanish-validated version of the scale was utilized (Nenclares and Jiménez, 2005). The scale had a Cronbach’s alpha of 0.90 in the present sample of FMS patients.
(ii) *Pittsburgh Sleep Quality Index* (Buysse et al., 1989). This scale consists of 19 items that are scored on a four-point Likert scale. Scale items assess subjective sleep quality in the past month based on seven components: subjective sleep quality, sleep latency, sleep duration, habitual sleep efficiency, extrinsic sleep disturbances, use of sleeping (hypnotic) medication, and daytime dysfunction. Each component is scored from 0 to 3 and higher scores indicate a greater degree of sleep impairment. The scale showed an internal consistency of 0.83 in the current study sample, who were administered the Spanish-validated version of the scale (Royuela and Macías, 1997).
(iii) *Epworth Sleepiness Scale* (Johns, 1991). This scale estimates subjective daytime sleepiness and comprises eight items. The scale assesses sleepiness in different daily situations and each item receives a score of 0–3 points. The Spanish-validated version of the scale (Chica et al., 2007) was used in the current study and the Cronbach’s alpha was 0.85.
(iv) *Sleep Impairment Index* (Morin, 1993). This scale comprises seven items that evaluate the subjective perception of the symptoms and consequences of insomnia, as well as how much the respondent is worried about these problems. Each of the seven items are scored on a four-point Likert scale and the total score ranges from 0 to 28. Higher scores indicate more severe insomnia. The scale showed a Cronbach’s alpha of 0.79 for the present sample of FMS patients that were administered the Spanish-validated version of the scale (Morin, 1998).

### Data Analyses

Due to the fact the data did not fit to a normal probability distribution, the *Mann–Whitney U*-test for independent samples was used to check for statistically significant differences in mean scores between allocation conditions. To test for statistically significant within-group differences between assessment phases, the *Friedman test* for *k* related samples was used and where the outcome was significant, the *Wilcoxon* test was subsequently employed. Bonferroni corrections were applied to lower the probability of finding a false significant difference due to chance. Cohen’s *d* and the percentage change between pre-test and post-test, as well as pre-test and follow-up scores were used to evaluate the magnitude of the change (where *d* = 1.5 or above reflects a very large effect-size, *d* = 1.5–1 reflects a large effect-size, and *d* = 1–0.5 reflects a medium effect size). All statistical analyses were conducted using SPSS version 22.0.

## Results

### Demographics, Completion, and Compliance

All participants were aged between 39 and 66 years (*M* = 51.8; *SD* = 10.2). A total of 8% of participants had no formal education, 62% had a primary-school education, 16% had intermediate studies education and 14% had a higher education. In terms of relationship status, 76% of participants were married, 14% were single, 3% were separated and 7% were widowed. All participants attended all assessment phases (i.e., there was no-missing data) and in respect of the at-home practice, the average rate of compliance was 71% for the body-scan exercise and 83% for the full awareness of breathing exercise.

### Intervention Effects

Means and standard deviations for each outcome measure for intervention and control group at all assessment phases are shown in **Table 1**.

**Table 1:** Means and standard deviations for each outcome measure at pre-test, post-test, and 3 month follow-up.

Variables	Pre-test		Post-test		Follow-up	
	*M*	*SD*	*M*	*SD*	*M*	*SD*
**Control Group**					
Subjective insomnia	14.2	4.7	14.7	4.7	13.9	3.9
Sleep quality	12.4	3.1	13.1	3.3	12.8	3.6
Sleepiness	15.6	3.4	15.9	4.6	16.1	4.4
Sleep impairment	16.9	5.9	17.2	5.8	16.3	6.1
**Intervention Group**					
Subjective insomnia	14.8	4.4	11.4	3.7	12.23	3.4
Sleep quality	13.0	3.9	9.1	3.3	10.37	3.1
Sleepiness	16.2	3.1	12.9	3.3	13.45	3.7
Sleep impairment	16.1	4.2	12.5	3.2	13.09	4.1

No significant baseline differences were observed between median scores for the intervention and control groups for any of the outcome measures. However, a significant difference was observed between groups at post-test for subjective insomnia, sleep quality, sleepiness, and sleep impairment. Furthermore, significant between group differences were observed at follow-up for subjective insomnia, sleep quality, sleepiness, and sleep impairment (see **Table 2**).

**Table 2:** Pre-test, post-test, and follow-up between-group differences for all outcome measures.

Variables	Pre-test		Post-test		Follow-up	
	*z*	*p*	*z*	*p*	*z*	*p*
Subjective insomnia	0.49	0.53	3.9	0.005^∗∗^	2.9	0.009^∗^
Sleep quality	0.32	0.64	4.0	0.005^∗∗^	3.4	0.006^∗^
Sleepiness	0.41	0.57	3.8	0.006^∗^	3.3	0.006^∗^
Sleep impairment	0.34	0.61	5.1	0.001^∗∗∗^	4.0	0.005^∗∗^

*^∗∗∗^p = 0.001; ^∗∗^p = 0.005; ^∗^p < 0.01.*

The *Friedman* test and follow-up *Wilcoxon* test showed that there were significant improvements across all outcome variables for the experimental group between pre-test and post-test, and between pre-test and follow-up. However, no significant difference was observed between post-test and follow-up for any of the outcome measures, suggesting that the reductions in sleep quality were maintained following conclusion of the mindfulness training (see **Tables 3**, **4**). No significant within-group differences were observed for the control group between any of the assessment phases.

**Table 3:** Friedman test for k related samples for each study variable in the intervention and control groups.

Group	Control		Intervention	
Variables	χ*2*	*P*	χ2	*P*
Subjective insomnia	1.3	0.51	10.7	0.008^∗^
Sleep quality	2.3	0.45	11.4	0.006^∗^
Sleepiness	1.9	0.48	12.7	0.003^∗∗^
Sleep impairment	2.9	0.39	13.4	0.001^∗∗∗^

*^∗∗∗^p = 0.001; ^∗∗^p < 0.005; ^∗^p < 0.01.*

**Table 4:** Wilcoxon test for related samples of the pre-test to post-test, pre-test to follow-up, and post-test to follow-up differences in the study variables in the intervention group.

Variables	Pre–Post		Pre–Follow-up		Post–Follow-up	
	*z*	*P*	*z*	*p*	*z*	*P*
Subjective insomnia	4.4	0.000^∗∗∗∗^	3.9	0.005^∗∗^	0.29	0.687
Sleep quality	5.1	0.000^∗∗∗∗^	3.6	0.007^∗^	0.57	0.416
Sleepiness	4.2	0.001^∗∗∗^	3.6	0.008^∗^	0.32	0.631
Sleep impairment	4.1	0.001^∗∗∗^	3.8	0.005^∗∗^	0.43	0.529

*^∗∗∗∗^p < 0.001; ^∗∗∗^p = 0.001; ^∗∗^p = 0.005; ^∗^p < 0.01.*

**Table 5** shows the effect size and percentage change between assessment phases for the intervention group. The pre-test to post-test change in the intervention group was of a large effect size for sleep quality (*d* = 1.07) and sleepiness (*d* = 1.02), and a medium effect size for sleep impairment (*d* = 0.93) and subjective insomnia (*d* = 0.84). For the change between pre-test and follow-up, medium effect-sizes were observed for sleepiness (*d* = 0.79), sleep quality (*d* = 0.76), sleep impairment (*d* = 0.71), and subjective insomnia (*d* = 0.65). Percentages of change in the aforementioned variables after the intervention were also important, ranging from 20 to 30% in the post-test phase, and from 17 to 20% at follow-up.

**Table 5:** Cohen’s *d* and percentage change in pre-test to post-test and pre-test to follow-up in study variables in the intervention group.

Variable	*d* Post-test	*d* Follow-up	% Post-test	% Follow-up
Subjective insomnia	0.8	0.65	-22.8	-17.4
Sleep quality	1.1	0.76	-29.7	-20.4
Sleepiness	1.0	0.79	-20.2	-16.9
Sleep impairment	0.93	0.71	-21.9	-18.4

## Discussion

The current study compared an SG-MBI known as *Flow Meditation* (*Meditación-Fluir)* with a waiting list control group to investigate the effects of mindfulness on sleep problems in female adults with FMS. Results showed that compared to the control condition, the mindfulness intervention was effective in reducing insomnia and improving sleep quality. To the authors’ knowledge, this is the first study to specifically investigate the effects of mindfulness on a range of sleep-related outcomes in individuals with FMS.

These findings are consistent with studies showing improvements in the number of hours of sleep in patients with depression after 8 weeks of mindfulness training (Britton et al., 2012; Kanen et al., 2015). Findings are also in line with studies demonstrating a reduction in general FMS symptomatology following mindfulness training (Schmidt et al., 2011; Langhorst et al., 2013; Parra and Latorre, 2013; Amutio et al., 2015a). Furthermore, findings are consistent with mindfulness studies focussing on conditions other than FMS that have shown reductions in pain-related psychological effects including sleep quality (Carlson et al., 2003; Day et al., 2012; Goyal et al., 2014; Henke and Chur-Hansen, 2014).

However, it should be noted that some studies of mindfulness (e.g., using mindfulness-based stress reduction – MBSR) have not demonstrated short-or long-term significant effects on sleep quality (Lauche et al., 2013). One possible explanation for this is that in the present study, sleep quality was thoroughly assessed via a battery of different sleep-relevant assessment tools that were able to capture a broader range of changes in overall sleep quality. A further explanation is that unlike MBSR that belongs to the first-generation of mindfulness approaches, the present study employed a mindfulness approach named *Meditación-Fluir* (Flow Meditation) that corresponds to a Second-Generation Mindfulness-Based Interventions (SG-MBIs; Van Gordon et al., 2016, 2017). Compared to first-generation mindfulness-based interventions, SG-MBIs employ a slightly different model of mindfulness that emphases the importance of non-attachment to self as well as to psychological and somatic symptoms (Van Gordon et al., 2015).

Although there is mixed-evidence and continued debate as to whether mindfulness decreases pain and improves quality of life in FMS patients (Schmidt et al., 2011; Henke and Chur-Hansen, 2014; Hervás et al., 2016), results of the present study offer additional support for the use of mindfulness to treat sleep problems in this patient group. In terms of possible mechanisms of action, poor sleep quality in individuals with FMS has been shown to negatively influence the regulation of emotion (Britton et al., 2012; Larson and Carter, 2016). Furthermore, abnormal sympathetic nervous system reactivity in patients with FMS is associated with variability in cardiac frequency during sleep (Prados and Miró, 2012). Additionally, pre-sleep arousal has also been found to be a partial mediator in the relationship between mindfulness and reported level of rest during sleep (Li et al., 2018). Therefore, in the present study, in addition to decreased hyperarousal of the sympathetic nervous system and increases in parasympathetic activation through relaxation, mindfulness may have improved sleep quality via the cultivation of new metacognitive resources (e.g., detachment from self-referential processing and acceptance) that increase perceptual distance from painful and/or distressing sensory and psychological stimuli (Lauche et al., 2013; Labelle et al., 2015; Van Gordon et al., 2016, 2017; Franco et al., 2017; Wheeler et al., 2017).

This is consistent with previous studies of *Meditación-Fluir* in which mindfulness was shown to improve state and trait anxiety, depression, and anger regulation in FMS patients (Amutio et al., 2015a) and in other populations including adolescents with high levels of aggressiveness and lack of impulse control (Franco et al., 2016) and older adults with high levels of anxiety, depression, and worry (Franco et al., 2017). These previous findings concerning improvements in anger regulation are particularly relevant since the use of adaptive coping strategies to recognize and express emotions has been associated with reduced burden of FMS (Castelli et al., 2012; Geenen et al., 2012; Sancassiani et al., 2017; Di Tella et al., 2018).

The beneficial outcomes observed in the present study were maintained for a minimum of 3 months. Similar maintenance effects have also been observed in other studies of mindfulness involving patients with FMS (Grossman et al., 2007; Amutio et al., 2015a; Cash et al., 2015; Van Gordon et al., 2017), as well as in studies following a similar approach to the model of mindfulness training adopted by the *Meditación-Fluir* intervention used in the present study (Amutio et al., 2015a; Franco et al., 2017). Some plausible explanations as to why the effects of mindfulness training programs are frequently maintained at follow-up assessments is that mindfulness is a technique that enhances positive affect and that it is relatively easy to integrate into daily life (Davis and Zautra, 2013; Amutio et al., 2015b; Galvez-Sánchez et al., 2018). This is an important consideration for patients with FMS that due to pain-related concentration deficits can experience difficulties in adapting to new psychological strategies (Schmidt et al., 2011; Van Gordon et al., 2016).

The findings of the present study should be considered in light of their limitations that include: (i) a small sample size, (ii) the absence of an active control condition, and (iii) reliance on self-report measures. Furthermore, although the study included a 3 month follow-up assessment, it would be useful to investigate maintenance effects over a longer period of time.

Fibromyalgia syndrome is a complex, multifaceted condition, which means that it is difficult to determine the precise components and functional mechanisms of mindfulness that are most active during its treatment. However, given the association between sleep quality and FMS symptomatology, and given the body of evidence suggesting that mindfulness can improve sleep quality in both clinical and non-clinical samples, it is reasonable to assume that improvements in sleep quality elicited through mindfulness play an important role in ameliorating symptomatology in individuals with FMS. This is in line with findings from this study, as well as with calls for FMS treatments that acknowledge the role of sleep problems in FMS and thus seek to directly alleviate this aspect of the condition (Theadom et al., 2007; Prados and Miró, 2012; Adler-Neal and Zeidan, 2017; Li et al., 2018).

The current study showed that a SG-MBI known as *Meditación-Fluir* was effective for reducing sleep problems in adults with FMS. While pharmacological treatments may lead to symptom reduction in FMS patients, some of these medications can cause disruptions in the physiology of sleep leading to adverse effects such as dizziness, headaches, and sleepiness (Britton et al., 2012; Prados and Miró, 2012). Consequently, non-pharmacological treatments, such as mindfulness, that helps to regulate and improve sleep quality, appear to have a role in the treatment of FMS. Thus, further research is warranted to replicate the findings of this study as well as understand the mechanisms that lead to improvements in sleep quality following participation in mindfulness training by individuals with FMS.

## Author Contributions

AA wrote and edited the different versions of the manuscript and the final paper. CF conceived and designed the study, and conducted data analysis. LS-S wrote the first draft of the paper. MP-F literature review and edited the final manuscript. JG-L literature review and corrections. MM-J bibliography and corrections. WVG collaborated in the writing and editing of the final manuscript.

## Conflict of Interest Statement

The authors declare that the research was conducted in the absence of any commercial or financial relationships that could be construed as a potential conflict of interest.
